# The Medical Profession and the New Taxes

**Published:** 1915-10-02

**Authors:** 


### THE MEDICAL PROFESSION AND THE NEW TAXES.

According to a very much over-worked saying, doctors are not good men of business, and any attempt at a financial article in a medical journal would probably be dismissed with a smile by the experts. But being flesh and blood like their neighbours, doctors know that when the bill comes in it has to be paid, and if they have assented to the bargain they are not likely to quarrel with the price. We take it that the great majority of our medical readers—probably, indeed, the whole of them—are at one with the nation on the justice of the nation's cause in the present war, and as they have willed the end they doubtless have willed also the means. Among these means is a necessary supply of money, and we are, therefore, as ready as our fellow-citizens to take our share in its provision. Even if we had the ability to criticise the financial scheme of the Budget we have no disposition so to do. The responsible authorities who alone know all the facts have given their decision, and, in the circumstances, it is for good citizens to pay up and—if possible—to look pleasant.

On schemes intended to promote economy and the avoidance of luxuries the medical profession is inclined naturally to turn an encouraging eye. The experience is common which shows that generally the more plain the living the more satisfactory the health, and though we are far from the position of those who would rob life of its reasonable pleasures and would erect weights and a balance on every dinner-table, we are satisfied that there is need for the encouragement of simplicity. In other words, the moderation and self-denial encouraged by some of the Chancellor's new proposals are measures which will tend to improve health rather than to prejudice it. In this direction, therefore, the proposals are likely to receive professional endorsement.

Some of the new taxes can hardly touch the medical profession. The super-tax has its home beyond our range, and "war profits" in medicine must be sadly to seek. Nor do the times admit of new motor-cars or bicycles, and to plate-glass and cinema films we have but little inclination. "Hats" might at one time have sounded a severe note, for the immaculate "topper" was in its day indispensable. But times have changed, and a looser fashion permits a more elementary and more economical headgear. In these respects "our withers are unwrung." For the rest we mostly suffer as our fellows. Income tax, in its various fashions and degrees, touches most of us, and for its increased severity we must try to extract consolation from the knowledge that while the tax is greater our incomes are less. Hence some modest revenge will come on the three years' average, and in any event the money is to be spent in a good cause. Those who have disciplined themselves in abstinence from alcohol may feel disappointed at the absence of a direct reward, but there is still the chance of saving on tobacco, not to speak of tea, coffee, cocoa, chicory, and dried fruits. Exhortations to patients in some of these directions are not unknown, and the opportunity for example remains open, and may now be blended with a sense of economic achievement. Towards an added threepence on motor spirit it is less easy to be philosophic, for here we suffer in a special degree. The position, however, is not without relief, at least in the Metropolis. For, so report runs, the omnibus fares are not to be increased, and there will be a special spice of pleasure in the thought that our own personal saving leaves the companies with the conviction that their petrol costs them more. After all, too, is not walking recommended by the highest authorities? Over the disappearance of the halfpenny postage there seems little occasion for tears if it will diminish the curse of the circular and enable us to economise on waste-paper baskets. Nor can we join in the groans of the 300 makers or vendors of patent and proprietary medicines who lament that "sick persons" will be called upon to bear the new burden, but whose anxiety does not prompt them to bear it themselves. To pass on the tax is doubtless a good commercial expedient, but it hardly jumps with a profession of grief for the customer. "I weep for you, the Walrus said, I deeply sympathise"—but we know what happened to the oysters.

